# What Controls the Sulfur Isotope Fractionation during Dissimilatory Sulfate Reduction?

**DOI:** 10.1021/acsenvironau.2c00059

**Published:** 2023-01-03

**Authors:** Min Sub Sim, Dong Kyun Woo, Bokyung Kim, Hyeonjeong Jeong, Young Ji Joo, Yeon Woo Hong, Jy Young Choi

**Affiliations:** † School of Earth and Environmental Sciences, Seoul National University, Seoul08826, Korea; ‡ Department of Earth and Environmental Sciences, Pukyong National University, Busan48513, Korea

**Keywords:** sulfate concentration, isotope effect, dissimilatory sulfate reduction, electron donor, temperature, phylogeny, cell-specific sulfate reduction rate

## Abstract

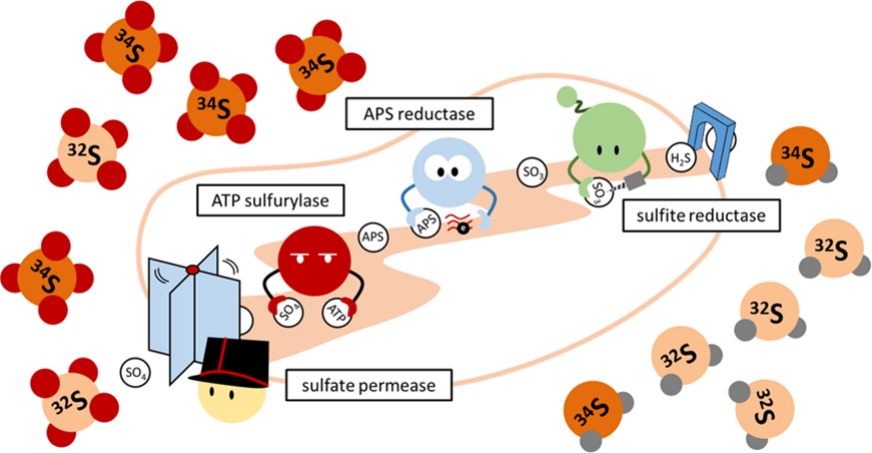

Sulfate often behaves conservatively in the oxygenated environments but serves as an electron acceptor for microbial respiration in a wide range of natural and engineered systems where oxygen is depleted. As a ubiquitous anaerobic dissimilatory pathway, therefore, microbial reduction of sulfate to sulfide has been of continuing interest in the field of microbiology, ecology, biochemistry, and geochemistry. Stable isotopes of sulfur are an effective tool for tracking this catabolic process as microorganisms discriminate strongly against heavy isotopes when cleaving the sulfur–oxygen bond. Along with its high preservation potential in environmental archives, a wide variation in the sulfur isotope effects can provide insights into the physiology of sulfate reducing microorganisms across temporal and spatial barriers. A vast array of parameters, including phylogeny, temperature, respiration rate, and availability of sulfate, electron donor, and other essential nutrients, has been explored as a possible determinant of the magnitude of isotope fractionation, and there is now a broad consensus that the relative availability of sulfate and electron donors primarily controls the magnitude of fractionation. As the ratio shifts toward sulfate, the sulfur isotope fractionation increases. The results of conceptual models, centered on the reversibility of each enzymatic step in the dissimilatory sulfate reduction pathway, are in qualitative agreement with the observations, although the underlying intracellular mechanisms that translate the external stimuli into the isotopic phenotype remain largely unexplored experimentally. This minireview offers a snapshot of our current understanding of the sulfur isotope effects during dissimilatory sulfate reduction as well as their potential quantitative applications. It emphasizes the importance of sulfate respiration as a model system for the isotopic investigation of other respiratory pathways that utilize oxyanions as terminal electron acceptors.

## 1 Introduction

Sulfur comprises about 2% of the Earth’s mass, being the sixth most abundant element on our planet,^1^ and exists in a wide range of oxidation states from sulfide (−II) to sulfate (+VI) in the Earth’s surface environments. Its abundance and multiple valence states make it an essential player in microbial redox processes such as anaerobic photosynthesis and respiration.^2^ Sulfate is quantitatively the most abundant oxidant in seawater but behaves nearly conservatively under oxygenated conditions as abiotic sulfate reduction is kinetically inhibited below 200 °C.^3^ In the deeper, anoxic layer of marine sediments, however, anaerobic respiration coupled to sulfate reduction accounts for more than half of the organic matter remineralization.^4^ Dissimilatory sulfate reduction is thus a fundamental driver for the redox cycling of sulfur at physiological temperatures; otherwise the marine sulfur cycle would be dominated by precipitation and dissolution of sulfate minerals. A metabolic product of dissimilatory sulfate reduction, sulfide, reacts with iron and forms insoluble pyrite, some of which can be incorporated and retained in sedimentary rocks over geologic time. Such burial of sulfur reduced at the expense of oxygenically produced organic carbon leaves dioxygen behind in the ocean and atmosphere.^5^ Therefore, changes in the biogeochemical sulfur cycle, largely representing the flow of sulfur between the oxidized, soluble sulfate and the reduced, less soluble sulfide, have interacted extensively with the redox cycle of other elements, including the evolution of atmospheric oxygen.^5,6^

Sulfur has four stable isotopes, ^32^S (95.04%), ^33^S (0.75%), ^34^S (4.20%), and ^36^S (0.015%),^7^ and as a major regulator of the global sulfur cycle, dissimilatory sulfate reduction shapes the patterns of sulfur isotope distribution in the Earth’s surface environments. Instead of absolute abundance, the sulfur isotope compositions are conventionally reported with the delta notation in per mil variations relative to the Vienna Canyon Diablo Troilite (VCDT): δ^X^S = 1000 × (^X^R_sample_/^X^R_VCDT_ – 1), where ^X^R is the ^X^S/^32^S ratio (X = 33, 34, or 36). Unless specified otherwise, in this minireview, sulfur isotope fractionation refers to the variations in the abundance of the two most abundant isotopes, ^32^S and ^34^S (^34^ε). Because the sulfur–oxygen bond containing heavier sulfur isotopes requires slightly more energy to break compared to the bond with ^32^S,^8,9^ dissimilatory sulfate reduction discriminates against heavy isotopes, producing sulfide depleted in ^34^S relative to the reactant sulfate by as much as 66‰.^10^ Compared to sulfate reduction, oxidative transformations of sulfur compounds involve relatively minor isotope fractionation from −6 to +12‰.^11^ Since first reported by Thode et al.,^12^ thus, sulfur isotope fractionation between sulfate and sulfide has been extensively used as a diagnostic marker for dissimilatory sulfate reduction in modern and ancient environments. Studies of the deep biosphere hundreds of meters below the seafloor^13^ and the billions-year-old marine sedimentary rocks^14^ are such examples. Recently, the sulfur isotope systematics on Mars and other extraterrestrial bodies have also emerged as a valuable tool for the search for potential biological signatures.^15^

In addition to its qualitative diagnostic value, dissimilatory sulfate reduction shows quantitatively a large range of sulfur isotope fractionation, ranging from −3 to +66‰.^10,16^ Since respiratory sulfur isotope fractionation is a phenotypic response to environmental factors via biochemical systems, an up to 70‰ variation ensures that sulfur isotope signatures in nature can capture not only the presence but also physiological information on sulfate reducing microorganisms. Importantly, the microbially fractionated sulfur isotope ratios locked in minerals are more stable and retained in sedimentary rocks over longer time scales than most constituents of biochemical systems such as polynucleotides and proteins, which makes them attractive in a variety of applications to reconstruct the past microbial activities and environmental conditions. To link the magnitude of isotope fractionation to the environmental parameters such as temperature, pH and availability of sulfate, electron donor and other key nutrients, over 800 sulfur isotope measurements have been made in more than 50 species of sulfate reducing microorganisms (Supplementary Table 1 and references therein). In the past decade, individual enzymes and intracellular metabolites involved in dissimilatory sulfate reduction have begun to be isotopically examined in order to assess the biochemical mechanisms underlying the sulfur isotopic response to environmental stimuli.^17−19^ In this minireview, we summarize such progress that has been made over the last half-century and highlight the remaining gaps in our understanding of the sulfur isotope fractionation during sulfate respiration.

## 2 Pathway and Sulfur Isotope Fractionation Model

**Figure 1:**
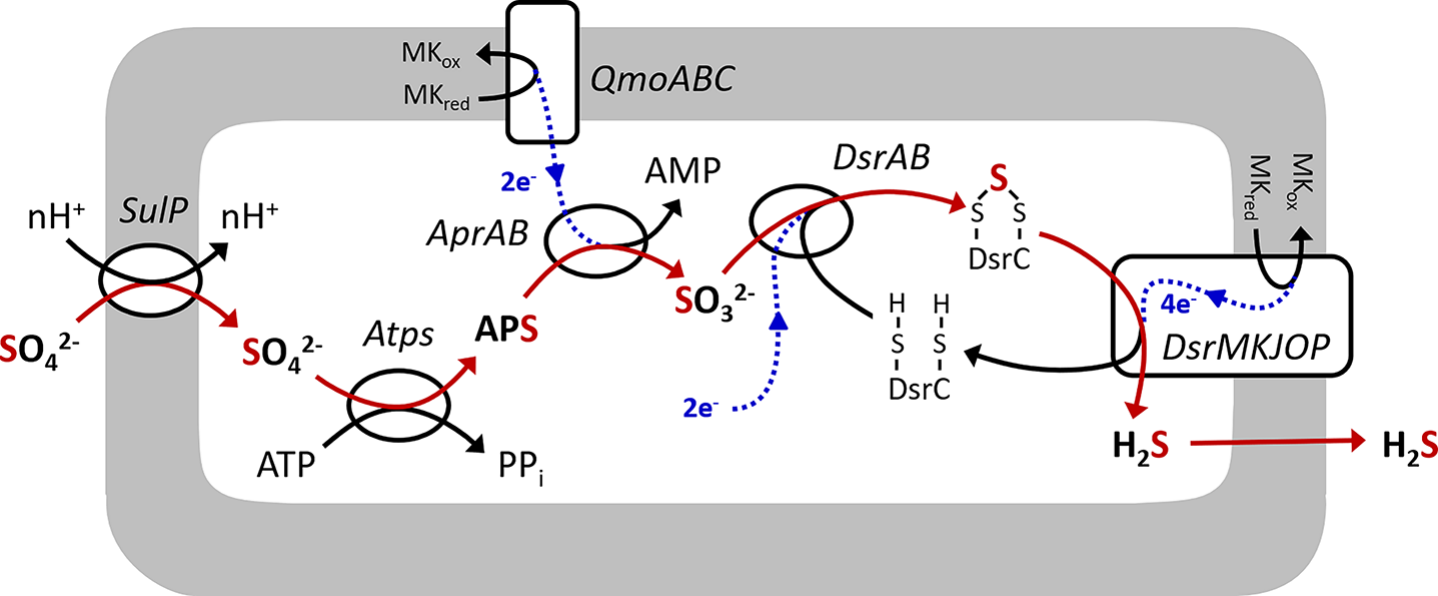
Sulfur metabolites and enzymes in the dissimilatory sulfate reduction pathway (modified after ref 23). Central enzymes implicated in sulfate respiration are sulfate permease (SulP), ATP sulfurylase (Atps), APS reductase (Apr), and dissimilatory sulfite reductase (Dsr). QmoABC and DsrMKJOP donate electrons for the reduction of APS and sulfite, respectively. MK_red_ and MK_ox_ stand for the reduced and oxidized forms of menaquinone.

Dissimilatory reduction of sulfate to sulfide involves four enzymes: sulfate permease (SulP), sulfate adenylyltransferase (Sat), adenosine phosphosulfate (APS) reductase (Apr), and dissimilatory sulfite reductase (Dsr) (Figure 1). First, the uptake of the negatively charged sulfate ion occurs in symport with either H^+^ or Na^+^ ions.^20^ Since sulfate is relatively inert under physiological conditions, it needs to be activated to APS prior to reduction at the expense of ATP hydrolysis, and then APS is reduced to sulfite using two electrons from the membrane-bounded QmoABC complex.^21^ The final step of sulfate respiration begins with the two-electron reduction by DsrAB. It had been debated whether the complete reduction to sulfide involves thiosulfate (S_2_O_3_^2–^) and trithionate (S_3_O_6_^2–^) as intermediates,^22^ but Santos et al.^23^ demonstrated that sulfite reduction by DsrAB produces the trisulfide form of DsrC, which is further reduced to sulfide and the thiol form of DsrC by the membrane DsrMKJOP complex.

**Figure 2:**
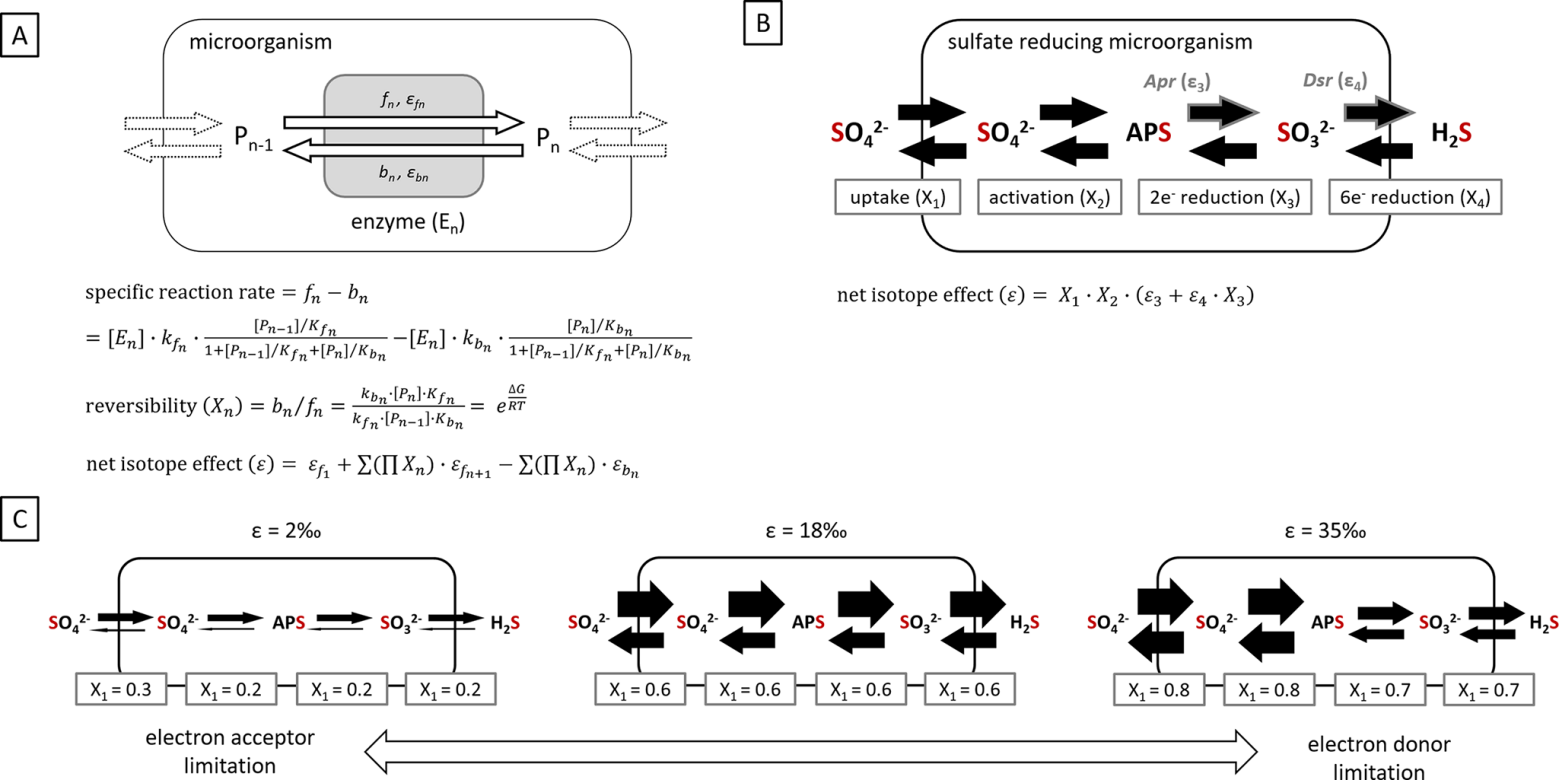
Schematic illustrations of the isotope fractionation in a series of reversible enzymatic steps. (A) In a quasi-steady state, the net reaction rate is equal to the difference between the forward (*f*) and backward (*b*) fluxes given by Michaelis–Menten kinetics, where [*E*] is enzyme concentration, *k* is the rate constant, [*P*] is the concentration of each metabolic intermediate, and *K* is the half-saturation constant. The net isotope fractionation (ε) is determined by the reversibility of the individual steps (*X*) in the reaction sequence and the kinetic isotope effects of each metabolite flux. The reversibility is an exponential function of the Gibbs free energy of reaction (Δ*G*), where *R* is the ideal gas constant and *T* is the absolute temperature. (B) The enzymatic pathway of dissimilatory sulfate reduction consists of four principle steps from sulfate uptake to the final reduction to sulfide. The mathematical expression for the total sulfur isotope effect by a cell can be simplified by assuming that the breakage of sulfur–oxygen bonds discriminates against heavy sulfur isotopes to a far greater extent than other steps. (C) A generalized mechanism for how changes in both electron donor and acceptor availability could influence the reversibility of rate-limiting enzymatic reactions and the magnitude of isotope fractionation. APS and sulfite reduction steps are assumed to fractionate the ^34^S/^32^S ratio by 20‰ and 50‰, respectively.

These four enzymatic reactions primarily shape the patterns of sulfur isotope fractionation during the dissimilatory sulfate reduction. In a quasi-steady state, where the concentrations of reaction intermediates remain approximately constant, the total isotope fractionation by any reversible, multistep enzymatic reaction can be expressed as a function of the isotope effects of the enzymes involved and the relative rates of each enzymatic reaction (Figure 2A). Since earlier isotope-labeling experiments showed that dissimilatory sulfate reduction is reversible as a whole,^24^ the reversibility of the SulP, Sat, Apr, and Dsr reactions and their kinetic isotope effects have served as the foundation for theoretical models for dissimilatory sulfur isotope fractionation, although the details have been modified to accommodate new findings.^22,25,26^

In this scheme, the sulfur isotope effects assigned to each enzymatic reaction constrained the potential range of microbial sulfur isotope fractionation. Until recently, kinetic isotope effects at the different steps have been assigned principally considering the results of culture experiments and equilibrium calculations.^22,25^ For example, sulfate uptake has been assumed to produce a small inverse sulfur isotope effect^25^ because Harrison and Thode^16^ reported that the isotope fractionation is inverted with ^34^SO_4_^2–^ reacting faster than ^32^SO_4_^2–^ at very low sulfate concentrations, where an increasing number of symported cations limits the backward flow across the membrane.^27^ There is a general consensus that large sulfur isotope effects are associated with the breaking of sulfur–oxygen bonds, but it was in the past decade that the kinetic isotope effects of the two reductive enzymes in the dissimilatory sulfate reduction pathway were directly constrained. The Apr, isolated from *Desulfovibro vulgaris*, fractionates the ^34^S/^32^S ratio by 20‰,^19^ which is 5‰ smaller than previously inferred.^25^ Although a sulfur isotope effect of 53‰ has been predicted for the entire Dsr reaction to explain the observed sulfur isotope fractionations,^22^ Leavitt et al.^17^ reported a fractionation of 15‰ for the first two-electron reduction by DsrAB, isolated from *D. vulgaris* and *Archaeoglobus fulgidus*, suggesting that the following reactions involving cytosolic DsrC and membrane-bound DsrMKJOP probably have a strong preference toward lighter sulfur isotopes.

Within the range defined by enzymatic sulfur isotope effects, it is the ratio of forward and backward reaction rates at each step of the dissimilatory sulfate reduction pathway that leads to variations in sulfur isotope fractionation at the cellular level (Figure 2A and B), and this flexibility provides a potential for the microbial sulfur isotope fractionation to respond to the environmental conditions that microorganisms encounter. The reversibility of each reaction step is governed by the Gibbs free energy of the reaction:
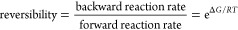
1where *R* is the gas constant, *T* is the absolute temperature, and Δ*G* is the free energy change associated with the reaction. Since the value of Δ*G* strongly depends on the concentrations of reactants and products, intracellular metabolite levels and their determinants like specific respiration rates, substrate and waste concentrations, and activity and kinetic properties of the involved enzymes should shape the patterns of sulfur isotope fractionation.^26^ Although a quantitative calibration of the model parameters requires a better understanding of the relevant enzymes and the intracellular biochemical processes, current models predict that either sulfate uptake or APS reduction acts as a metabolic bottleneck in the sulfate respiration pathway under physiological conditions.^26^

## 3 Factors responsible for Variability of Isotope Fractionation

### 3.1 Temperature

**Figure 3:**
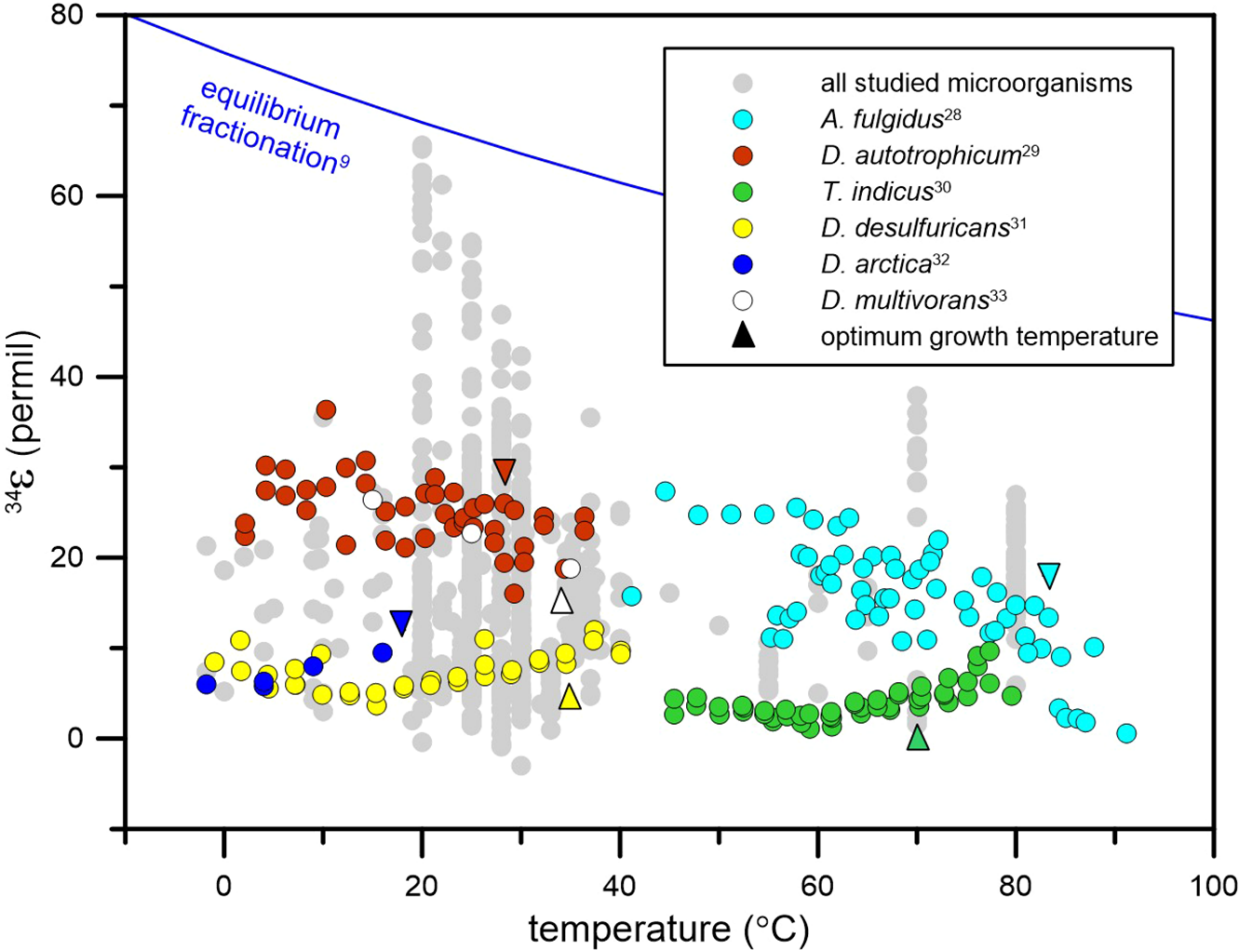
Effect of temperature on ^34^S/^32^S isotope fractionation during dissimilatory sulfate reduction in pure culture studies. There is no universal correlation between temperature and isotope effect, but published enrichment factors are smaller than the equilibrium values at the given temperature.

Temperature is an environmental variable that directly influences biological processes and thus has been studied as a determinant of the microbial sulfur isotope fractionation. While whole cell studies have shown that fractionation of sulfur isotopes varies considerably with temperature (Figure 3), no temperature dependence of enzymatic sulfur isotope effect exceeding the experimental errors has been observed for Apr and Dsr.^17,19^ Marked differences in the responses to temperature between enzyme- and cellular-level fractionations demonstrate that the reversibility, not the kinetics of individual enzyme, plays a key role in explaining large variations in dissimilatory sulfur isotope fractionation. Given the divergent results of previous culture studies (Figure 3), however, it is unlikely that the reversibility of each reaction changes uniformly with temperature. In studies where the sulfur isotope fractionation spanned around 20‰, the magnitude of fractionation decreased with increasing temperature,^28,29,33^ but the response was inverted when the overall fractionation did not exceed much beyond 10‰.^30−32^ Although there could be multiple explanations for the conflicting observations, one possibility is that higher temperatures augment biochemical processes at key metabolic bottlenecks. Sulfur isotope effects of a few permil require that sulfate transport into the cell barely exceeds the downstream demand,^18^ and enhanced sulfate uptake with increasing temperature would lead to greater sulfur isotope discrimination (Figure 2B and C). On the other hand, the cellular-level fractionation comparable to that of Apr (20‰) suggests that Apr acts as the metabolic bottleneck.^19^ In that case, faster APS reduction at higher temperatures would limit the backward reaction from APS to sulfate, reducing the sulfur isotope fractionation (Figure 2B and C). Thus, the relationship between temperature and isotope fractionation varies largely depending on the relative contribution of individual reactions to the overall reversibility of the sulfate respiratory pathway.^31,33^ However, a 10‰ change in sulfur isotope fractionation corresponds to variations in temperature greater than 25 °C (Figure 3), while the naturally occurring fractionation ranges over 70‰;^19^ temperature unlikely acts as a predominant control on sulfur isotope partitioning over most of the Earth’s surface environments.

A notable exception would be the underwater hydrothermal fields, where a highly dynamic interface between cold, oxygenated seawater and hot, reduced hydrothermal fluids results in steep temperature and geochemical gradients. At such elevated temperatures, not only microbial kinetic isotope effects but also equilibrium isotope fractionation between sulfate and sulfide changes considerably (Figure 3). Since backward reactions through the sulfate reduction pathway have been assumed to fractionate sulfur isotopes negligibly,^22,25^ the maximum isotope fractionation attained by dissimilatory sulfate reduction is expected to approach the equilibrium fractionation and thus to decrease as temperature increases. Indeed, no laboratory culture studies have shown the sulfur isotope effect greater than equilibrium values at the given temperature (Figure 3), but in deep ocean sediments at elevated temperatures, apparent sulfur isotope fractionations often exceed their equilibrium values.^34^ This discrepancy suggests that further work is needed to refine our understanding of the sulfur isotope fractionation by thermophilic microorganisms and their role in the hydrothermal environments.

### 3.2 Sulfate Concentration

**Figure 4:**
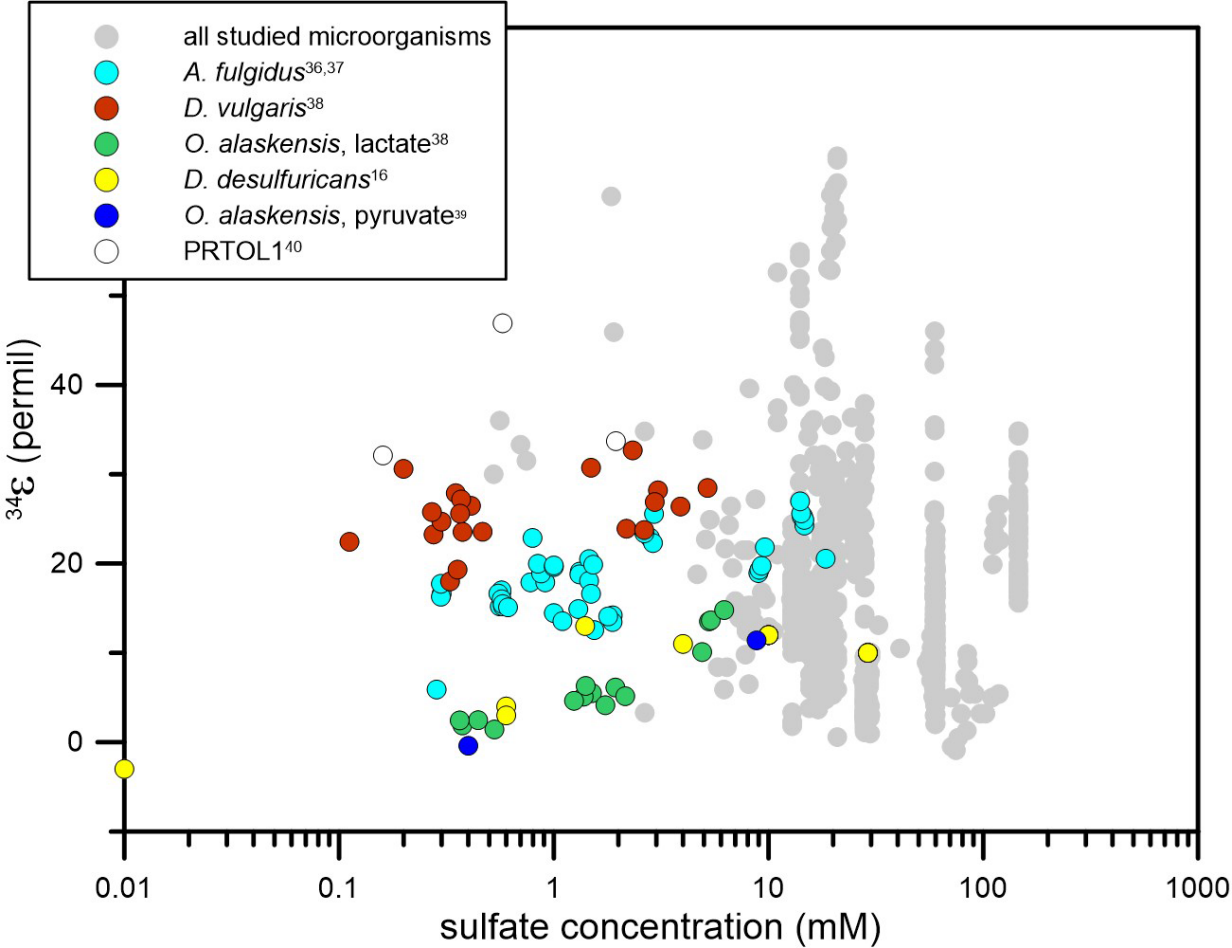
Compilation of published data on the dissimilatory sulfur isotope effects as a function of sulfate concentration. Submillimolar sulfate concentrations have been shown to generally decrease the magnitude of isotope fractionation.^39,40^

Sulfate is a fundamental prerequisite for dissimilatory sulfate reduction, but its availability varies markedly between different habitat types and also throughout geologic time. For example, sulfate is the most abundant dissolved electron acceptor in modern seawater, where its concentration is as high as 28 mM, while most terrestrial environments contain only micromolar concentrations of sulfate. Also, the sulfate concentrations in the Precambrian oceans appear to have been more than an order of magnitude lower than the that of modern seawater.^35^ The impact of sulfate availability on microbial sulfur isotope fractionation, therefore, has been of great interest as a potential indicator of environmental sulfate levels. An early pioneering work showed that at the concentrations given by the solubility of the sparsely soluble sulfates, the magnitude of fractionation declined and even reversed in sign as ^34^SO_4_^2–^ was consumed slightly faster than ^32^SO_4_^2–^ (Figure 4).^16^ These findings were further validated by sulfate-limited continuous culture studies,^36−38^ confirming that depletion of the terminal electron acceptor, sulfate, leads to smaller sulfur isotope effects (Figure 4).

Muted isotopic fractionation under sulfate limitation can be attributed to less reversible sulfate uptake. Basically, sulfate permease functions in a reversible manner, but its reversibility changes depending on the transport stoichiometry.^18,27,41^ Given the symport system with proton (or sodium ion) as a coupling ion, the free energy available from proton inflow is a primary driving force of sulfate uptake, and sulfate reducing microorganisms increase the proton/sulfate stoichiometry of sulfate transport in response to a decrease in the external sulfate concentration, accumulating sulfate inside the cell.^41^ An increasing number of symported protons makes sulfate uptake more energetically favored and less reversible, thereby reducing the isotopic fractionation. Because sulfate transport is the first step of dissimilatory sulfate reduction pathway, its reduced reversibility effectively nullifies the isotope effect of reductive enzymes in the downstream of the pathway (Figure 2B and C); that is, if all transported sulfate is quantitatively reduced to sulfide, no further isotopic discrimination can occur inside the cell.

Both experimentally and theoretically, there is a strong consensus that sulfate limitation tends to decrease the magnitude of fractionation, but the required threshold concentration of sulfate appears to vary with species and growth conditions. Habicht et al.^36^ demonstrated that less than 200 μM sulfate suppressed the isotopic fractionation by *A. fulgidus*, a hyperthermophilic archaeon, but in later work using *D. vulgaris*,^38^ sulfur isotope fractionation at 100 μM sulfate remained as large as it was at 6 mM sulfate (∼25‰). Relatively large isotope fractionation exceeding 20‰ has been also reported from natural habitats with micromolar concentrations of sulfate.^42,43^ Thus, although sulfate availability is one of the crucial factors governing the magnitude of fractionation, sulfur isotope compositions may not provide a unique calibrator for ambient sulfate concentrations.

### 3.3 Electron Donor

**Figure 5:**
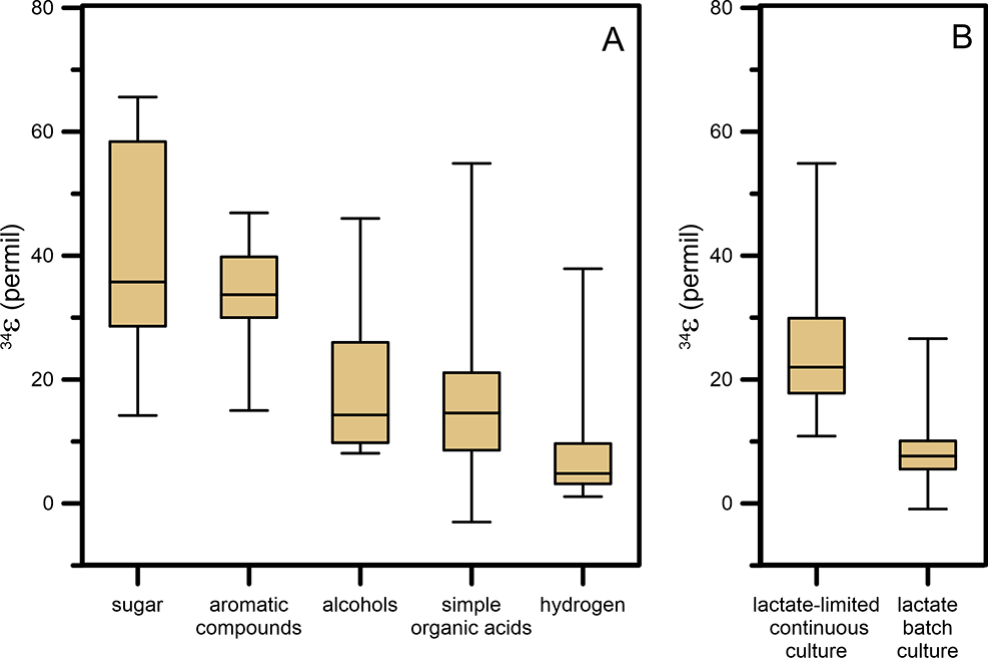
Effect of electron donors on the ^34^S/^32^S fractionation by sulfate reducing microorganisms. (A) Box-and-whisker plot of the published sulfur isotope data as a function of the type of electron donors. (B) Even with lactate as a sole electron donor, dissimilatory sulfur isotope effects differ significantly depending on its availability.

Dissimilatory sulfate reduction requires an electron donor, usually in the form of organic compounds, and in most marine habitats, sulfate respiration is primarily regulated by organic substrate supply rather than sulfate availability as sulfate concentrations in marine sediments are usually well above the half-saturation concentration for sulfate reduction.^44−46^ The role of electron donors in sulfur isotope fractionation has been extensively studied with pure cultures of dissimilatory sulfate reducers, demonstrating that the limitation by electron donors or the presence of recalcitrant organic substrate increases the magnitude of isotopic fractionation.^16,47−51^ Although there are some variations across species, sulfate reduction coupled to the oxidation of molecular hydrogen or simple organic acids such as lactate produced a relatively small isotope effect, while a larger sulfur isotope effect was obtained during the growth on carbohydrates or aromatic substrates (Figure 5). With lactate as a sole electron donor, however, growth under lactate-limited conditions in a continuous culture increases the sulfur isotope discrimination between sulfate and sulfide up to over 50‰ (Figure 5),^51^ highlighting that large isotope fractionation can occur even in the presence of labile substrates if they are present in a limited quantity. For the last two decades, it has been also shown that a single species of a sulfate reducing bacterium grown with different electron donors fractionates ^34^S/^32^S ratio from 6‰ to 66‰, spanning almost the entire range of isotopic fractionation occurring in nature.^10,50,52^

A marked influence of the electron donor on the magnitude of sulfur isotope discrimination can be manifested by changes in the reversibility of the Apr and Dsr reactions (Figure 2B and C). Both scarcity and reduced reactivity of electron donors likely shift the intracellular redox balance toward a more oxidizing state, which will lead to a less negative free energy change of APS and sulfite reduction and make these reactions more reversible (eq 1). There is currently no experimental data on variations in the intracellular redox state, determined by redox pairs such as NAD^+^ and NADH, corresponding to the changing isotopic fractionation. However, a deficiency in the electron transport system induced by mutation or iron limitation^53,54^ and diverting electron flow to the alternative pathway such as nitrogen fixation^53^ indeed increase the sulfur isotope fractionation during sulfate respiration, corroborating that the redox balance of the electron transfer chain determines the reversibility of sulfate reduction pathway.

Contrasting effects of sulfate and electron donors on the isotopic fractionation show that a degree of balance between these two catabolic processes is fundamental to understanding a wide range of sulfur isotope fractionation in nature. For example, smaller sulfur isotope fractionation prevails in freshwater and terrestrial environments with micromolar sulfate concentrations, while larger isotope fractionations are more common in deep sea sediments characterized by the limited availability and the poor reactivity of organic substrates (refs (10, 51, 55) and references therein). It is noteworthy, however, that the present-day seawater SO_4_^2–^ concentration of 28 mM guarantees that the effect of electron donors on the sulfur isotope distribution in marine environments greatly exceeds that of sulfate as it would have been throughout the most Phanerozoic period.^56^

### 3.4 Cell-Specific Sulfate Reduction Rate

**Figure 6:**
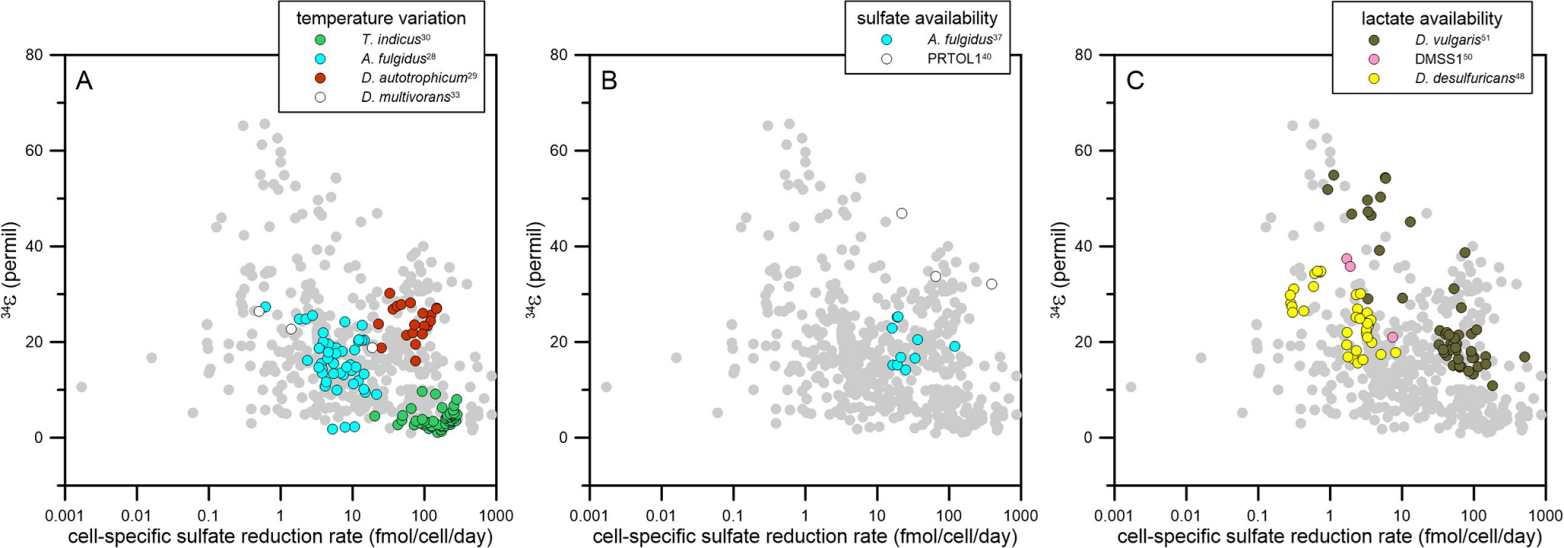
Sulfur isotope fractionation as a function of cell-specific sulfate reduction rates in pure cultures. Each panel highlights the variation induced by changes in temperature (A), sulfate concentration (B) and lactate concentration (C).

Since Harrison and Thode^16^ demonstrated that *Desulfovibrio desulfuricans* fractionated ^34^S/^32^S ratio to a greater extent as the rate of sulfate reduction decreased, an inverse relation between the isotope effect and the cell-specific sulfate reduction rate (csSRR) has been described in several pure culture studies,^30,47−51^ which has been often used to predict the rate of sulfate reduction in the modern and ancient environments.^38,51^ The csSRR in pure culture studies covers a range of 0.002–860 fmol SO_4_^2–^/cell/day, and with the exception of the two slowest rates reported from retentostat experiments,^57^ the maximum fractionation reached at the given csSRR decreases rapidly as the rate increases, whereas the minimum value remains less than 10‰ (Figure 6). Different species shows a varying relationship, and experiments with different temperatures or sulfate concentrations do not always show the inverse trend between csSRR and isotope effect, resulting in the broad scatter of the combined data set (Figure 6). However, the correlation becomes more significant when results are compared within a single species, in particular for the experiments on the role of electron donors. For example, limiting the lactate supply in the continuous culture of *D. vulgaris* increases the isotope effect from 10‰ to over 50‰, while decreasing the csSRR by 2 orders of magnitude (Figure 6).^51^ Similar to the amount of the electron donor, its reactivity also affects both parameters in a correlated manner.^50^

Since the in situ rate of sulfate reduction in marine environments is dependent on the amount of the organic matter present in sediments and its availability to microbial degradation,^58−60^ the inverse relationship presented by the electron donor experiments can provide a useful constraint on the marine sulfur cycle. However, this correlation is not readily apparent from a metabolic modeling scheme, which attributes the varying magnitude of fractionation to the reversibility of each enzymatic step (Figure 2A). In the steady-state, csSRR is equal to the difference between the forward and backward fluxes in any steps of the sulfate reduction pathway, and both forward and backward reaction rates, given by Michaelis–Menten kinetics, are proportional to the enzyme concentration. In contrast, the enzyme concentration is canceled out in the calculation of the ratio between the two fluxes (reversibility) (Figure 2A). Theoretically, therefore, increasing the concentration of respiratory enzymes could increase the rate of sulfate reduction, while leaving the reversibility unchanged or even increased. Although this hypothesis has not been tested experimentally so far, it is interesting to note that a faster csSRR is accompanied by a larger isotopic fractionation during diazotrophic growth.^53^ Since nitrogen fixation acts as an additional sink for electrons, while increased energy demand necessitates a faster respiration rate, the complexity inherent in the relationship between csSRR and isotope effect could be resolved by the comparison of respiration-related gene expression between diazotrophic and nondiazotrophic conditions. Thus, sulfur isotope fractionation may generally reflect the specific rate of sulfate reduction in marine habitats, but it should be kept in mind that its causality warrants further investigation.

Also noteworthy is that the largest sulfur isotope fractionation was observed around the center of the measured range of the csSRR (Figure 6). At the lower end of the range, where a minimum csSRR is required for the maintenance energy, isotopic fractionation would be small as highly reversible reactions creating futile cycles of sulfur metabolites may not support growth.^54,61^ On the other hand, since a simultaneous increase in both csSRR and isotope effect requires higher concentrations of respiratory enzymes (Figure 2A), the upper right quadrant of Figure 6 may represent an extreme, perhaps physiologically unrealistic scenario.

### 3.5 Phylogeny

Although all known sulfate reducing microorganisms share a common dissimilatory pathway which involves the activation of sulfate to APS and the reduction of APS to sulfite and finally to sulfide,^62^ this capability is patchily distributed in both bacterial and archaeal phyla, and the enzymes primarily responsible for sulfur isotope fractionation, Apr and Dsr, have a complex evolutionary history including events of gene duplication and horizontal transfer.^63,64^ Thus, in addition to the physiological controls on the magnitude of isotopic fractionation, there could be inherent phylogenetic differences between the microorganisms. In the fractionation model outlined in Figure 2, an obvious question about the phylogenetic effect is whether the kinetic isotope effects of the enzymes, namely Apr and Dsr, vary significantly among different lineages or not. So far, the sulfur isotope effect of AprAB has been determined only for a single species, *D. vulgaris*,^19^ but bacterial and archaeal DsrAB isolated from *D. vulgaris* and *A. fulgidus*, respectively, have shown a similar in vitro sulfur isotope effect of 15‰.^17^ While deserving further investigation across a wider phylogenetic range of taxa, these initial findings may suggest an overall similarity in the enzymatic isotope effect as the order and sequences of the aprAB and dsrAB genes are highly conserved among sulfate reducing microorganisms.^63,64^

**Figure 7:**
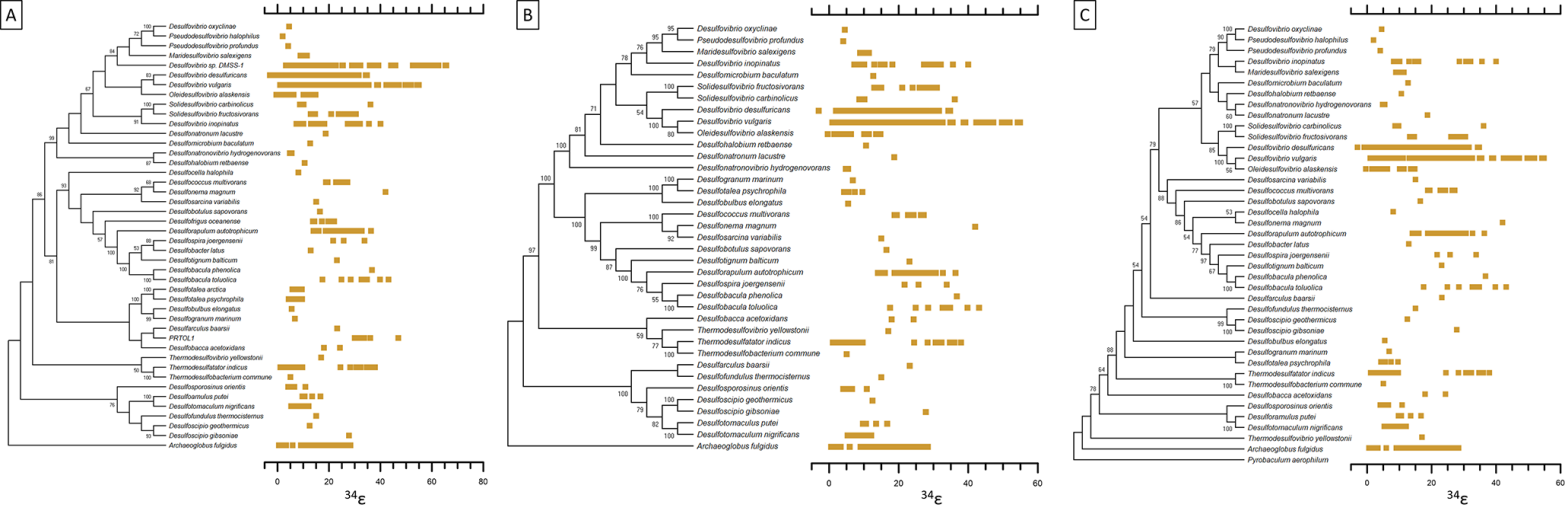
Patterns of the sulfur isotope fractionation in comparison to the phylogenetic tree of sulfate reducing microorganisms based on 16S rRNA (A), AprA (B) and DsrB (C) sequences. The trees were constructed using MEGA X software with the maximum likelihood method. Numbers before each branch point represents the percentage of bootstrap resampling based on 100 trees. Bootstrap values below 50% are not shown. Accession numbers for all sequences are given in the Supplementary Table 1.

At a cellular level, Detmers et al.^65^ examined the relationship between the phylogenetic distance based on the 16S rRNA gene sequences and the magnitude of sulfur isotope fractionation, revealing no significant correlation across the 32 investigated sulfate reducers. Rapid advances in molecular biology over the last two decades have allowed us to assess the distribution of sulfur isotopic phenotypes in different phylogenetic lineages based on the gene sequences that encode the reductive enzymes, Apr and Dsr, as well as rRNA (Figure 7). Regardless of the gene used for phylogenetic analysis, however, the patterns of microbial sulfur isotope fractionation are not apparently related to the topology of phylogenetic tree. A few species belong to the *Desulfovibrio* genus, such as *D. vulgaris* and DMSS1, appear capable of fractionating the isotope ratios to over 40‰, and fairly comparable results have been obtained for distantly related species, including the thermophilic *Thermodesulfatator indicus* (Figure 7). Many other species within the same clade with *D. vulgaris*, however, do not produce such large sulfur isotope effects (Figure 7). Moreover, although disseminated over the whole phylogenetic tree, isotopic fractionations greater than 40‰ are always associated with either limited supply or reduced reactivity of electron donors (Supplementary Table 1 and references therein). Our phylogenetic analysis of the expanded data set with more taxa and genes thus confirm the previous conclusion that the sulfur isotope fractionation by microorganisms is largely dependent on their physiology rather than the phylogenetic positions.^65^ More specifically, it is the redox state of sulfate reducing microorganisms, but not the subtle structural variation between homologous enzymes, that has a greater influence on the changes in sulfur isotopic phenotypes.

## 4 Concluding Remarks and Perspectives

Over 50 years of laboratory experiments on dissimilatory sulfur isotope effects, in parallel with the development of theoretical models for sulfate reduction, have revealed that the relative accessibility of sulfate and electron donors strongly controls the magnitude of fractionation, and as this balance shifts to the oxidative side, energetically less favorable and more reversible reduction allows for larger sulfur isotope effects.^26,38^ This relationship has been extrapolated to environmental levels and provided a qualitative measure of the sulfate reducing activity in marine sediments,^51,66,67^ the largest habitat for sulfate reducing microorganisms, where the rate of sulfate reduction usually depends on the amount of organic matters and their availability for biogenic degradation.^59,60^ The use of sulfur isotopes as a quantitative indicator is however rather complex, in part because the reversibility of multiple steps cannot be uniquely resolved from the fractionation of the ^34^S/^32^S ratio (Figure 2), and second because the relationships between the magnitude of fractionation and environmental parameters vary across taxa. Although challenging, advances in stable isotope mass spectrometry, complemented by biochemical approaches, help to address these two issues.

First, using isotope ratios other than the traditional ^34^S/^32^S could provide additional constrains on the reversibility of each reaction step. Sulfur has multiple stable isotopes (^32^S, ^33^S, ^34^S, and ^36^S), and while the fractionation of four sulfur isotopes exhibits a power law mass dependency, a linear mixing of different sulfur pools along the sulfate reduction pathway and a slight difference of mass-dependent effects in sulfur isotope fractionation processes make both ^33^S/^32^S and ^36^S/^32^S ratios measurably deviate from the single step low-temperature equilibrium trends.^68−70^ These variations do not exceed a few tenth of permil but carry unique information independent from that obtained in conventional δ^34^S analysis. The distribution of the metabolic fluxes through the dissimilatory sulfate pathway can be further illustrated by oxygen isotope measurements. Enzymatic reductions should lead to a shift in the ^18^O/^16^O ratios of residual sulfate toward higher values as is in the case of δ^34^S, while the oxygen isotope exchange between intracellular water and sulfite can decouple the isotope effects on sulfur and oxygen.^71,72^ Thus, a relationship between δ^34^S and δ^18^O values reflects the balance between the rate of sulfate reduction and the rates of isotope exchange and sulfite reoxidation.^72,73^ Using ^17^O-labeled water, the role of oxygen isotope exchange can be assessed independently from the mass-dependent ^18^O/^16^O fractionation,^74^ and recently, ^34^S–^18^O clumping in sulfate also emerges as a promising tool to resolve the individual components of the microbial sulfate reduction pathway.^75,76^ Nevertheless, this theoretically promising approach requires isotopic characterization of the involved enzymes for quantitative analysis.^50,52^ To date, the triple sulfur isotope effect of DsrAB^17^ is the only available data on the multiple sulfur isotope fractionation imposed by the enzyme in the sulfate reduction pathway, and neither oxygen isotope effect nor rare isotope clumping has been investigated. Further enzymatic studies are thus warranted to establish a firm basis for a quantitatively calibrated model that links the magnitude of fractionation to their biological controls.

Second, physiological differences between sulfate reducing microorganisms may elicit differing sulfur isotopic responses to the same environmental changes. For example, within the genus *Desulfovibrio*, submillimolar sulfate concentrations decrease the sulfur isotope fractionation by the marine sulfate reducing bacteria *Desulfovibrio alaskensis* down to a few permil, whereas the freshwater *D. vulgaris* fractionates sulfur isotopes by more than 20‰ under the same condition.^38^ It is, however, worth noting that the reversibility of the overall pathway and thereby the sulfur isotope effect are the function of intracellular metabolite levels and biochemical parameters such as membrane proton gradient, phosphorylation potential and intracellular redox state.^19,26,77^ Although the sulfate reduction pathway is highly conserved,^62^ metabolic pathways to translate various environmental stimuli into intracellular biochemical signals are complex and more species-specific. Therefore, the magnitude of fractionation associated with sulfate respiration, if it is to be a quantitative indicator, needs to be calibrated against the intracellular redox and phosphorylation potentials, rather than directly against environmental parameters.

Finally, given that dissimilatory sulfate reduction is one of the most intensively studied metabolisms in the field of isotope geochemistry, the concepts discussed here and further progress in understanding the sulfur isotope fractionation along the respiratory pathway can be extended into other dissimilatory pathways that utilize oxyanions as terminal electron acceptors.^78−80^

## Supporting Information Available

Literature compilation of the kinetic isotope effects during sulfate reduction together with growth conditions, cell-specific sulfate reduction rates, and nucleotide accession numbers (XLSX)
